# Antimicrobial Nanoplexes meet Model Bacterial Membranes: the key role of Cardiolipin

**DOI:** 10.1038/srep41242

**Published:** 2017-01-25

**Authors:** Alejandro Marín-Menéndez, Costanza Montis, Teresa Díaz-Calvo, Davide Carta, Kostas Hatzixanthis, Christopher J. Morris, Michael McArthur, Debora Berti

**Affiliations:** 1 Procarta Biosystems Ltd, Norwich Innovation Centre, Norwich, UK; 2 Department of Chemistry “Ugo Schiff” and CSGI, University of Florence, Firenze, Italy; 3 Norwich Medical School, University of East Anglia, Norwich, UK; 4 School of Pharmacy, University of East Anglia, Norwich, UK

## Abstract

Antimicrobial resistance to traditional antibiotics is a crucial challenge of medical research. Oligonucleotide therapeutics, such as antisense or Transcription Factor Decoys (TFDs), have the potential to circumvent current resistance mechanisms by acting on novel targets. However, their full translation into clinical application requires efficient delivery strategies and fundamental comprehension of their interaction with target bacterial cells. To address these points, we employed a novel cationic bolaamphiphile that binds TFDs with high affinity to form self-assembled complexes (nanoplexes). Confocal microscopy revealed that nanoplexes efficiently transfect bacterial cells, consistently with biological efficacy on animal models. To understand the factors affecting the delivery process, liposomes with varying compositions, taken as model synthetic bilayers, were challenged with nanoplexes and investigated with Scattering and Fluorescence techniques. Thanks to the combination of results on bacteria and synthetic membrane models we demonstrate for the first time that the prokaryotic-enriched anionic lipid Cardiolipin (CL) plays a key-role in the TFDs delivery to bacteria. Moreover, we can hypothesize an overall TFD delivery mechanism, where bacterial membrane reorganization with permeability increase and release of the TFD from the nanoplexes are the main factors. These results will be of great benefit to boost the development of oligonucleotides-based antimicrobials of superior efficacy.

Due to the rising incidence of antimicrobial resistance in hospitals and the paucity of new therapeutics in the pipeline^1^ there is renewed interest in innovative approaches such as oligonucleotide therapeutics. Besides the selectivity towards their therapeutic target, there are no known mechanisms by which bacteria could expel oligonucleotides outside their cytoplasm, as they do with small molecule antimicrobials via drug efflux pumps^2^,^3^,^4^, as proved, e.g., for the bacterial pathogen *Escherichia coli* (*E. coli*)^5^. However, though promising, antisense molecules, which block or prevent translation of mRNA, are effective only at micromolar concentrations. This is due to a combination of factors such as the difficulty of delivery and the high cytoplasmic concentration of their target mRNA molecules. Transcription Factor Decoys (TFDs) are oligonucleotide copies of the DNA-binding site for transcription factors. When transfected into bacterial cytoplasm they competitively inhibit transcription of essential genes: a single TFD blocks the synthesis of thousands of copies of mRNA, explaining its greater potency. Thus, TFDs are established tools and potential therapeutics in the dissection and treatment of several human diseases^6^. We were the first to demonstrate their utility in bacteria to control gene expression^7^ with decoy oligonucleotides. One of the main hurdles that still hamper full exploitation of this approach is the delivery to bacterial cytoplasm. In the case of the Gram-negative pathogens, such as *E. coli*, this involves crossing four different biological barriers: a lipopolysaccharide (LPS) outer layer and two orthogonal membranes sandwiching a thin peptidoglycan layer. Moreover, the charged TFDs need to be encapsulated in a complex to protect them from degradation and rapid clearance in biological fluids. In this respect, nanoparticle formulations where DNA is complexed thanks to electrostatic compensation, such as cationic polymers^8^, lipids/liposomes^9^, or self-assembling systems as bolaamphiphilic molecules, have been widely investigated. Bolaamphiphiles are surface-active molecules with two hydrophilic end groups connected by a hydrophobic chain. So far, a large variety of bolaamphiphiles has been synthesized and the study of their self-organizing and packing properties led to the generation of new insights into the biophysics of membranes as well as to the creation of novel bolaamphiphiles^10^,^11^,^12^. Recently, a novel bolaamphiphilic molecule, 12,12′-(dodecane-1,12-diyl)bis(9-amino-1,2,3,4tetrahydroacridinium), hereafter termed 12-bis-THA, a dequalinium-derivative (Fig. 1a), has been described. It forms nanoplexes, which selectively target bacteria, therefore offering a potentially bacteria-selective gene-delivery system^13^. We propose that targeting of non-redundant transcription factors in bacterial cells is less likely to lead to resistance compared to conventional antibiotics and antimicrobial peptides^14^,^15^ which can induce minor mutations and profound phenotypic shift in target bacteria^16^. Resistance to cationic antimicrobials with structural similarity to 12-bis-THA (e.g. quaternary ammonium compounds and biguanides) is associated with the expression of plasmid-encoded efflux pumps such as qacA^17^. In contrast, our approach uses nanoparticles based on 12-bis-THA designed to facilitate the entry of TFDs through subtle membrane perturbation rather than serving as a direct membrane-disrupting anti-bacterial.

In this study we investigated the bacterial delivery mechanism of a TFD, designed to specifically inhibit the SigH transcription factor in *Clostridium difficile* (*C. difficile*), (the hairpin structure of the TFD is sketched in Fig. 1b) encapsulated in 12-bis-THA nanoplexes. The efficient delivery of the TFD inside bacteria and the fulfillment of its biological task, i.e., the inhibition of essential gene transcription, are mainly hampered by the bacterial cell capsule that impedes the access to exogenous species. Thus, a thorough comprehension of the delivery mechanism and of the main factors affecting the delivery efficiency is a necessary prerequisite for the design, development and improvement of TFD-based antimicrobials of superior efficacy. In order to investigate the interaction pathway of nanoplexes with bacteria, we employed *E. coli* and *C. difficile* as model organisms for pathogenic bacteria and liposomes of variable composition as model synthetic membranes, providing complementary information contributing to establish fundamental knowledge on the delivery mechanism. *E. coli* is characterized by a well understood structure of the cell capsule as of the composition of the two phospholipid membranes (cytoplasmic and outer). The outer membrane, which is the greater barrier to delivery, contains three major phospholipids: the zwitteronic Phosphatidylethanolamine (PE, typically 80% by mass), the anionic Phosphatidylglycerol (PG, 15%) and Cardiolipin (CL, 5%) lipids, which ratios vary as a function of cell cycle^18^ temperature^19^ and even amongst regions of the cell wall^20^,^21^,^22^. Though the lipid compositions also vary among other bacteria, PG and CL phospholipids are always present but are crucially absent in eukaryotic plasma membranes. Many studies highlighted a peculiar membrane distribution of CL, which preferentially accumulates in areas of greater curvature, such as the septum and poles of mycobacteria^23^ and *E. coli*^24^,^25^ and forms CL-domains in *E. coli* and *B. subtilis*^26^,^27^. Consequently, it was suggested that bacterial membranes contain a mosaic of microdomains of different lipids^28^ with a polar and septal accumulation of CL that may ensure proper spatial segregation and distinct biological functions including roles in cell division and respiration^29^,^30^. Synthetic lipid membranes, such as Giant Unilamellar Vesicles^31^,^32^, Supported Lipid Bilayers^33^,^34^ or Monolayers^35^ and Liposomes^36^,^37^,^38^, have been generally recognized as valuable synthetic mimics to investigate cell membrane-related phenomena as diverse as raft-like domains formation, membrane proteins behavior, membrane fusion and cell trafficking. The opportunity to tune and control the composition, curvature and structure of a model membrane system affords the mechanistic dissection and modeling of binding events at complex *in vivo* biomembranes. In our study we combined Confocal Microscopy data on TFD-12-bis-THA nanoplexes delivered to *E. coli* bacteria, with Fluorescence and Dynamic Light Scattering data on the interaction of the same nanoplexes with liposomes containing different relevant lipids present in the bacterial membranes. Our results provide valuable insights in the TFD delivery mechanism to bacteria, and in particular on the key role played by the CL molecules accumulated in specific regions of the bacterial membranes. Moreover, preliminary biological efficacy and toxicity data are presented, highlighting the promising features of the presented nanostructure system as antimicrobial therapeutic.

## Results and Discussion

**Figure 1:**
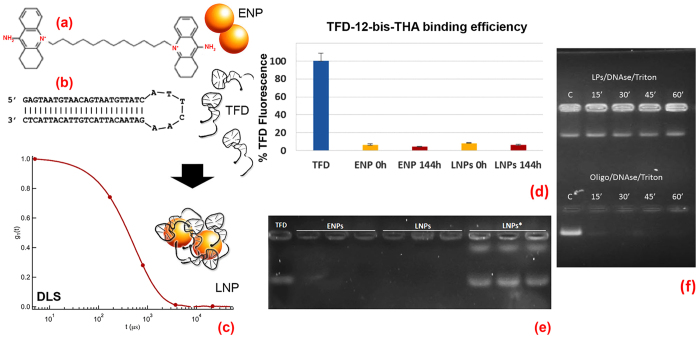
TFD-12-bis-THA nanosystem characterization. (**a**) Chemical structure of the bolaamphiphile 12-bis-THA, which self-assembles in water to give “Empty Nanoplexes” (ENPs); (**b**) Hairpin secondary structure of the oligonucleotide Transcription Factor Decoy (TFD); (**c**) Representative normalized DLS curves of 12-bis-THA complexed with the TFD, forming “Loaded Nanoplexes” (LNPs) prepared as described in the experimental section. (**d**) % Fluorescence intensity of the DNA intercalating agent SYBR Green excited at λ = 498 nm and acquired at λ = 522 nm in the presence of free TFD (set at 100%), ENPs and LNPs at 0 h and 144 h after preparation; (**e**) Electrophoresis in 1% agarose gel of free TFD (lane 1), ENPs (lane 2–4), LNPs (lane 5–7) and LNPs* after treatment with Sodium Taurocholate to induce TFD release (lane 8–10). (**f**) DNAse protection assay. LNPs with N:P ratio 11 (12-bis-THA, 180 μM and TFD, 10 μg/ml) were prepared and incubated with 10 u/mL DNase I. Reactions were stopped at the indicated times by addition of EGTA.

The main components of the nanostructured antimicrobial are displayed in Fig. 1. The bolaamphiphile 12-bis-THA (Fig. 1a) is characterized by a saturated hydrophobic chain of twelve carbon atoms connecting two acridinium monopositive polar headgroups. Due to hydrophobic interactions, 12-bis-THA self-assembles in water at a 0.18 mM concentration, forming nanosized entities (named empty nanoplexes, ENPs)^13^ with very low scattering intensity, whose hydrodynamic diameter (DLS) and zeta potential was estimated around 180 nm and +43 mV, respectively. TFD (Fig. 1b) complexation by 12-bis-THA and the formation of nanoplexes, named loaded nanoplexes (LNPs), can be followed through DLS (a representative DLS curve of LNPs is displayed in Fig. 1c), highlighting the formation of particles of 180 ± 10 nm hydrodynamic diameter (0.25 PDI) and +27 ± 3 mV zeta potential, formed with 0.18 mM concentration of 12-bis-THA and 10 μg/mL TFD. The decrease in the positive charge of the NPs upon incubation with TFD (from +43 mV to +27 mV) and the simultaneous ten-fold increase of the scattering intensity with respect to ENPs, due to the electrostatic compensation between the 12-bis-THA positive polar headgroup and the polyanionic oligonucleotide, are clear evidence of TFD complexation. LNPs maintain a net positive surface charge, allowing an electrostatically driven interaction with the anionic bacterial membrane. The efficiency of TFD complexation by 12-bis-THA was evaluated through the fluorescence intensity of DNA intercalating agent, SYBR Green (Fig. 1d), and from agarose gel electrophoresis (Fig. 1e). In Fig. 1d the normalized fluorescence intensity of SYBR Green intercalated in the free TFD (set at 100%) is compared to that of LNPs measured 0 h and 144 h after sample preparation. The dramatic decrease of fluorescence intensity in LNPs (above 95%) can be attributed to a DNA hairpin compaction upon complexation that prevents SYBR Green intercalation. It is known that double helix compaction is a necessary prerequisite for the development of nucleic acid-based therapeutics, in order to protect the genetic materials from DNase degradation^39^,^40^. Moreover, TFD-12-bis-THA complexes appear stable, since the fluorescence intensity of SYBR Green is not recovered in a 144 h time span. The agarose gel image (Fig. 1e) compares the electrophoretic mobility of TFD alone (first lane) and inside LNPs (second-fourth lanes). Clearly, TFD electrophoretic mobility in LNPs is hampered, as no band attributable to released TFD can be detected in the gel for LNPs and the TFD is released only when the LNPs are broken by incubation with an emulsifier, sodium taurocholate (labeled with *, Fig. 1e). Protection against DNase degradation was also investigated by agarose gel electrophoresis (Fig. 1f). TFD complexed into LNPs was resistant to degradation up to 60 min (Fig. 1f, top). In contrast, TFD incubated with DNaseI in the absence of 12-bis-THA was undetectable on the gel at 15 min incubation time point and beyond (Fig. 1f, bottom) indicating rapid degradation. In summary, the structural characterization of the LNPs, thus, highlights the formation of stable particles of around 180 nm hydrodynamic diameter and net positive surface charge, where the TFD is complexed in a compacted conformation, remains stably associated with the LNP over a prolonged period of time and is not spontaneously released.

**Figure 2:**
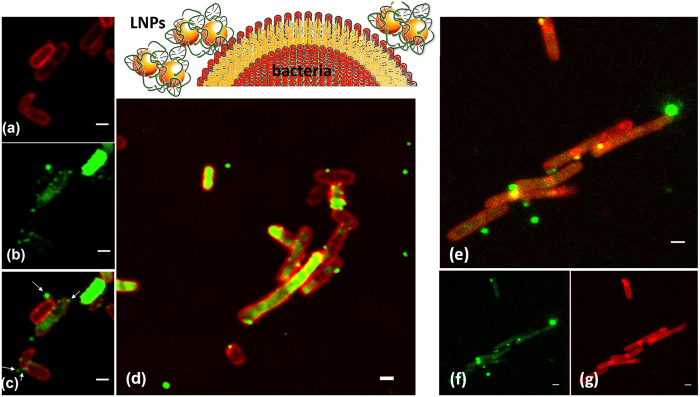
LNPs-bacterium interaction and TFD delivery: confocal microscopy. Representative CLSM images of (**a**–**d**) *E. coli* and (**e**–**g**) *C. difficile* bacteria labeled with the WGA-TMR and FM™ 4-64FX dye (red) on the membrane, respectively, and incubated with LNPs containing Alexa 488 labeled TFD (green) (**a**,**b**,**f**,**g**) separate channels and (**c**–**e**) merged channels displayed. Bar length 1 μm in all cases.

The expected mechanism of action of this antimicrobial nanosystem implies the contemporary destabilization and possible disruption of the bacterial membrane, combined with the dissociation of the LNPs to release the TFD inside the bacterial cytoplasm, where the oligonucleotide performs its specific antibacterial activity. In order to better understand these mechanistic aspects, we investigated the interaction of LNPs with two representative bacteria, *E. coli* as a model Gram-negative bacterium and *Clostridium difficile*, a Gram-positive pathogen. Figure 2 displays representative confocal microscopy (CLSM) images of (a–d) *E. coli* and (e–g) *C. difficile* treated with a fluorescent WGA-TMR dye for membrane staining challenged with LNPs containing Alexa 488-labelled TFD.

Following incubation of *E. coli* with nanoparticles, LNPs can be seen as bright green puncta colocalizing with the WGA-labeled cell periphery (see Fig. 2a–d) with several cells containing a diffuse green TFD signal intracellularly. The images were captured after 4 h but time course studies suggested uptake was rapid and occurred within 20 minutes. Clearly, from CLSM images a close interaction of the LNPs with the bacterial membrane is highlighted, probably driven mainly by the electrostatic attraction between the positively charged LNPs and the negatively charged bacterial membrane. However, from Fig. 2a,g specifically, the bacterial membranes appear intact using CLSM, although rearrangements might have occurred at the sub-microscopic level. Similar images were captured for the Gram-positive bacterium, *C. difficile* that has a markedly different cell wall structure- featuring a proteinaceous capsule, a thickened peptidoglycan layer and a single phospholipid membrane (Fig. 2e–g). For both bacteria the internalized TFD signal is diffuse, suggesting that the nanoparticles release their TFD cargo after crossing the cell walls, which is an important property for a transfection agent. Finally, it can be noticed that in several bacteria the LNPs are associated with poles and septa of the cells. These regions of the membrane are characterized by a higher curvature and, thus, by a peculiar lipid composition: in particular they are known to be enriched in Cardiolipin^41^ due to the overall conical shape of its headgroup that means it favors areas of high curvature^25^. The simultaneous presence of LNPs concentrated on the surface of the bacteria for some cells and of diffuse TFD fluorescence inside other cells, for the same samples, suggests that TFD delivery inside the bacteria occurs as two separate events, the first being a close interaction between the LNPs and the bacterial membrane and the second being the release of the TFD inside the cells’ cytoplasm, with a potential role of the CL in the overall delivery mechanism.

**Figure 3:**
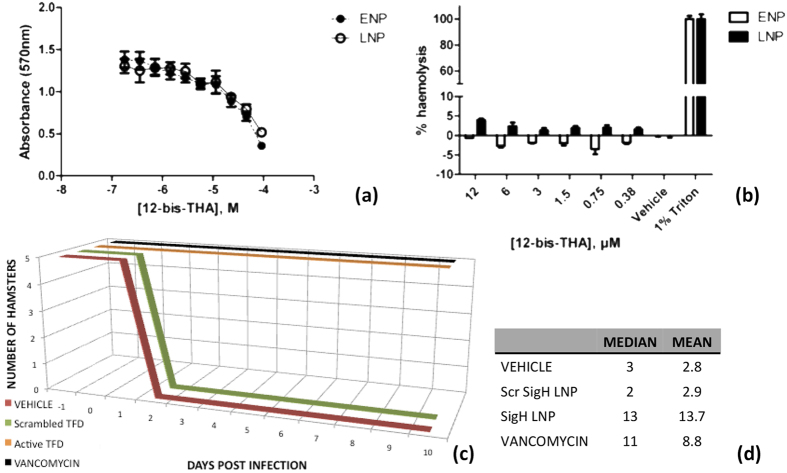
Biological properties of LNPs. (**a**) MTT assays were performed on Caco-2 cells after exposure to ENP/LNP for 24 hrs. mean ±SD; n = 6, (**b**) Haemolytic activity of ENP/LNP after 1 h exposure to fresh rat erythrocytes. Triton X-100 was +ve control (100% haemolysis) and HBSS (diluent) as biocompatible negative control, n = 3 ±SD. (**c**) LNPs loaded with SigH TFD were as efficacious in clearing a *C. difficile* infection of a Golden Syrian Hamster model as an ED_50_ dose of Vancomycin, whereas the vehicle control and an LNP loaded with a scrambled version of the TFD failed to rescue the animals; (**d**) Kaplan-Meier Survival estimates in days for each arm.

Biological assays were carried out in order to test the features of LNPs as possible antimicrobial drugs. A MTT assay was performed to verify their toxicity on eukaryotic cells (Fig. 3a), as compared to its antimicrobial activity. After 48 h incubation Caco-2 viability decreased in a dose-dependent manner with an IC_50_ of 61 ± 1 μM for ENP and 68 ± 2 μM for LNP. These values are indicative of modest toxicity potential of ENP/LNP. Low aqueous solubility of 12-bis THA prevented the preparation of NP samples at concentrations sufficient to cause toxicity comparable to the Triton X-100 positive control. This might compromise the accuracy of curve fitting of the MTT data and, as such, might overestimate cytotoxicity. As a further examination of biocompatibility rat erythrocyte haemolysis assays confirmed that both ENP and LNP have low haemolytic potential (Fig. 3b). At the highest concentration tested ENP caused no measurable haemolysis while LNP induced haemolysis was 3.9 ± 0.4%. The concentrations required to effect significant cytotoxicity are much higher than MIC values (typical MICs values are around 20 nM, on *E. coli*, data not shown). However, this suggests that the formulation could be employed for oral delivery to treat infections of the gut, while intravenous administration demands further work to decrease cytotoxic effects. The anti-bacterial efficacy of LNPs loaded with a SigH-targeted TFD to treat *C. difficile* infections was tested in a severe animal model established in Golden Syrian Hamsters (Fig. 3c,d). Briefly, the intestines of the animals were sterilized by clindamycin treatment prior to inoculation with a potentially lethal dose of spores. One hour later the treatments were started in groups of five animals. The vehicle arm, consisting of the buffer alone (50 mM MES pH 5.6) succumbed to the infection within 2 days as did the LNP containing a scrambled version of the SigH TFD at 2 mg/kg (Fig. 3c). The other two arms were the positive control (25 mg/kg vancomycin) and the SigH LNP (at 2 mg/kg) that both gave full protection until the end of the study and were statistically superior to the vehicle control (Fig. 3d). Post-mortem observations concluded there was no discernable difference between these two arms: for both there was some indication of slight inflammation in the small intestine and in most animals the caecum was somewhat enlarged. The microbiological burdens were consistent with the vancomycin and SigH LNP treatments effectively and rapidly killing vegetative *C. difficile* cells. Effectively by 24 h no cells were detected in the faecal sample, whereas in the vehicle or scrambled TFD LNP 1.67 × 10^5^ CFU/mL and 8.04 × 10^5^ CFU/mL were detected. Hence, SigH LNPs were effective in treating the infection by clearing vegetative bacteria, were well tolerated and showed no signs of toxicity.

**Figure 4:**
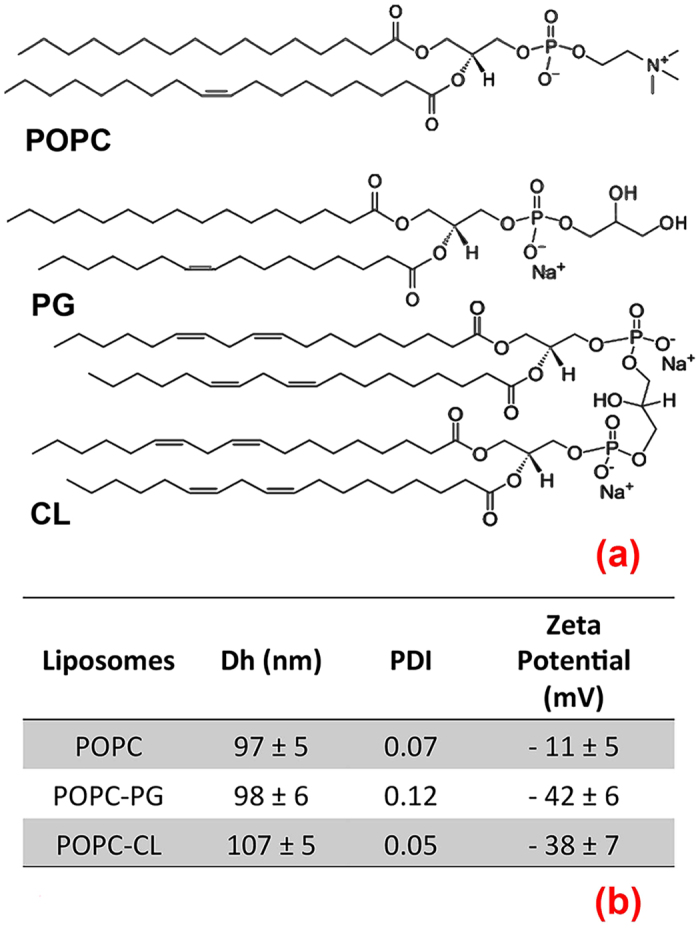
Membrane models. (**a**) Structure of the lipids employed in liposomal formulation: zwitterionic POPC (1-palmitoyl-2-oleoyl-*sn*-glycero-3-phosphocholine), monoanionic PG (L-α-phosphatidylglycerol (sodium salt)), dianionic CL (Cardiolipin (sodium salt)); (**b**) Physicochemical features of the liposomes: hydrodynamic diameter (Dh) and polydispersity (PDI) obtained from DLS and Zeta potential values of liposomes of bare POPC (POPC), of POPC:PG mixtures (42% PG w/w, POPC-PG) and of POPC:CL mixtures (42% CL w/w, POPC-CL) prepared as described in the experimental section.

Understanding the delivery mechanism will facilitate development of TFD-12-bis-THA-based therapeutics and, more in general, of antimicrobial agents of superior efficacy against antimicrobial resistance. To this end, we studied the interaction of LNPs with simplified models of bacterial membranes by studying liposomes with varying phospholipid content and hence, different physicochemical features. Liposomes were prepared with different ratios of three common lipids present in bacterial outer membranes: zwitterionic POPC (1-palmitoyl-2-oleoyl-*sn*-glycero-3-phosphocholine), monoanionic PG (L-α-phosphatidylglycerol (sodium salt)) and dianionic CL (cardiolipin (sodium salt)) (Fig. 4a). Zwitterionic liposomes composed entirely of POPC of around 100 nm hydrodynamic diameter and −11 ± 5 mV zeta potential (Fig. 4b) were compared to liposomes of similar size and net negative charge (around −40 mV zeta potential), containing high percentages, 42% w/w, of PG or CL (Fig. 4b). Thus, we were able to build-up simplified bacterial models characterized by same size but different surface charge, distinguishing POPC zwitterionic liposomes (slightly negative) from anionic POPC-PG and POPC-CL liposomes (highly negative, but with a similar overall surface charge between them). Moreover, while POPC and PG are common lipids, CL has a peculiar structure similar to two covalently bound PG molecules, implying a particularly high charge density and a non-zero spontaneous curvature^42^.

**Figure 5:**
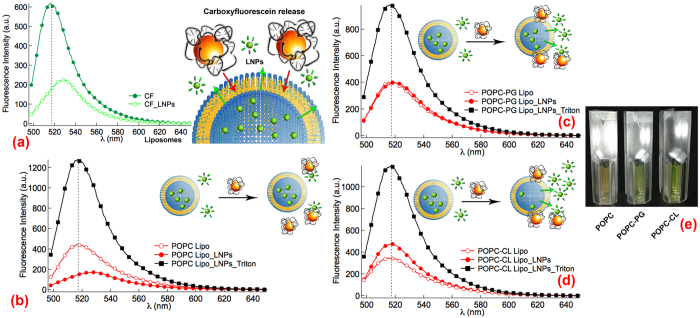
LNPs-liposomes interaction: carboxyfluorescein release. (**a**) CF quenching effect upon interaction with LNPs, as revealed from the fluorescence spectra measured upon excitation at λ = 488 nm; Scheme representing the experiment: permeability of the liposomal membrane upon interaction with LNPs is monitored through the dequenching of CF contained in the aqueous pool of liposomes; (**b**–**d**) fluorescence spectra of liposomes containing carboxyfluorescein 60 mM in the aqueous pool in the absence (empty red markers) and in the presence (filled red markers) of LNPs, and after addition of Triton X 100 1% (black markers) excited at λ = 488 nm, (**b**) POPC, (**c**) POPC-PG, (**d**) POPC-CL liposomes; (**e**) liposomes of different formulations containing 60 mM CF incubated with LNPs: cuvettes visualization upon illumination with white light.

As a first step, we investigated the effects of LNPs on lipid membrane destabilization-permeability. The three liposomal formulations were prepared with a self-quenching concentration (60 mM) of carboxyfluorescein (CF) in the aqueous pool^43^. Thus, liposomal leakage and membrane destabilization upon interaction with the LNPs can be followed through the increase in CF fluorescence intensity once released from the interior of the liposomes to the surrounding medium (as sketched in Fig. 5a). Figure 5b–d report some representative fluorescence spectra measured at λ_exc_ = 488 nm for bare POPC, POPC-PG and POPC-CL liposomes (red lines and empty markers), liposomes in the presence of 1% Triton X-100 (black lines and markers), liposomes in the presence of LNPs (red lines and filled markers). The fluorescence intensity measured for liposomal solutions can be attributed to the traces of CF outside of the liposomes that remain after the purification process. However, when the liposomes are disrupted upon addition of a strong detergent (1% Triton X-100 solution) to form mixed micelles, a three-fold increase of CF fluorescence intensity is observed, due to the complete release of the CF from the aqueous core of the liposomes and dilution of the fluorophore in the whole solution volume.

The presence of LNPs causes a strong decrease in intensity and a red shift of the maximum of the emission spectrum of CF in the zwitterionic POPC liposomal sample (Fig. 5b). This effect can be attributed to the interaction of the LNPs with the residual CF not encapsulated inside the liposomes. To prove this hypothesis, when CF is incubated with either ENPs or LNPs, a decrease in the fluorescence intensity of the peak at 515 nm is observed, together with a red shift of the fluorescence peak of approximately 10 nm, from 515 to 525 nm (Fig. 5a). This is due to the association of the positively charged 12-bis-THA and the di-anionic CF, which results in a change of the electronic properties of the fluorophore as reported for other fluorophore-aromatic amine pairs^44^. Therefore, for POPC liposomes the addition of LNPs does not lead to an increase of CF fluorescence intensity, which would indicate a CF release, but rather results in an overall decrease of CF fluorescence intensity, due to the interaction of LNPs with the residual CF in solution. Conversely, when POPC-PG liposomes were challenged with LNPs (Fig. 5c), neither decrease of CF fluorescence intensity, nor a redshift of CF fluorescence peak at 515 nm were detected. The overall effect, briefly sketched in Fig. 5c, can be attributed to the combination of two opposed processes, i.e., the interaction between the LNPs and the residual CF molecules in the external aqueous medium (which would lead to an overall decrease of CF fluorescence intensity) and the leakage of CF from POPC-PG aqueous pool, due to a strong interaction between the positively charged LNPs and the negatively charged lipid membrane (which leads to a net increase of CF fluorescence intensity). Finally, the addition of LNPs to POPC-CL liposomes (Fig. 5d) determines a net increase in CF fluorescence intensity, which clearly indicates for these samples the occurrence of CF release from the aqueous pool of liposomes, provoked by the interaction with the LNPs. The clear evidence of these data is the key role played by the anionic lipids in driving the interaction of LNPs with the liposomal membrane (representative images of CF release Fig. 5e). As already mentioned, a paramount step in the efficient delivery of nanoplexes relies in their ability to interact with and alter the functionality of the bacterial membrane. CF leakage highlights that the electrostatically-driven interaction between LNPs and anionic target membranes (POPC-PG and POPC-CL) determines a structural destabilization of the liposomal bilayer and an increased permeability that is not detected for the zwitterionic target membrane (POPC liposomes). In addition, a clear difference is detected for POPC-PG and POPC-CL membranes, despite of the overall identical net surface charge of the liposomal membrane. Therefore, the specific molecular properties of the CL with respect to PG, both in terms of surface charge density and of spontaneous curvature, are likely to play an important role in determining a stronger interaction of the LNPs with the target membrane. In particular, we can hypothesize that the electrostatically-driven interaction between the positively charged LNPs and the lipid bilayer, leads to an additional membrane destabilization when CL is present in the liposomal membrane, due to the conformational frustration of CL molecules, which are more prone to interact with the LNPs and, thus, to form transient pores in the membrane that allow membrane passage of small molecular species, such as CF.

**Figure 6:**
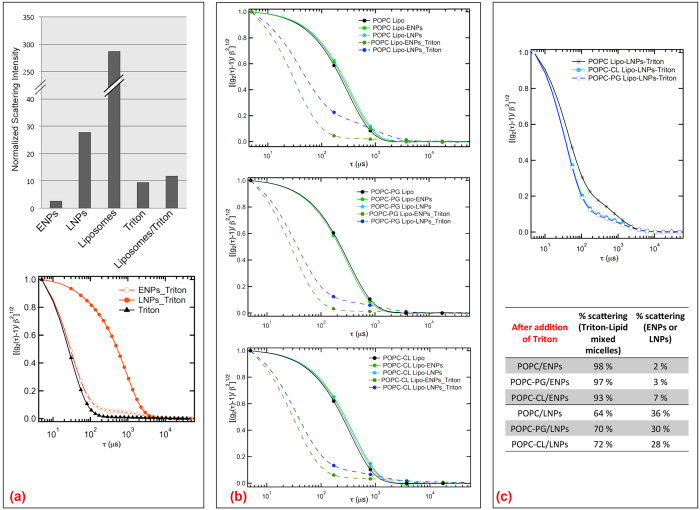
LNPs-liposomes interaction: Dynamic Light Scattering. (**a**) Histogram representing the normalized scattering intensity measured at 532 nm at 90° for ENPs, LNPs, Liposomes, Triton X100 and Liposomes in the presence of Triton X100 1% (Liposomes-Triton); Representative normalized ACF measured for 1% Triton (black markers) and for ENPs (orange empty markers) and LNPs (orange filled markers) in the presence of 1% Triton; (**b**) Representative normalized ACF measured for bare liposomes (black lines and markers), for liposomes in the presence on ENPs before (continuous green lines and markers) and after (dashed green lines and markers) the addition of 1% Triton and for liposomes in the presence on LNPs before (continuous blue lines and markers) and after (dashed blue lines and markers) the addition of 1% Triton; (**c**) Normalized ACF measured for POPC (black), POPC-PG (blue) and POPC-CL (light blue) liposomes in the presence of LNPs after addition of 1% Triton; Table summarizing the DLS data on liposomes/ENPs and liposomes/LNPs samples after addition of 1% Triton, as obtained from Laplace inversion with CONTIN algorithm, reported as relative contribution of the overall scattering intensity of the population of Triton mixed micelles (around 20 nm hydrodynamic diameter) and of ENPs or LNPs (around 200 nm hydrodynamic diameter).

In order to further evaluate the effects of ENPs and LNPs on the overall structure of liposomes, we performed a DLS study, whose results are reported in Fig. 6. The normalized (i.e., divided by the scattered intensity of toluene in the same experimental conditions) average scattered intensities at 90° of the different samples at the same concentrations as in the mixed samples (ENPs, LNPs, liposomes, 1% Triton, liposomes +1% Triton) are reported in Fig. 6a. Interestingly, ENPs and LNPs are characterized by a very different intensity that can be attributed to a different local structure of the scattering objects, which leads to a different scattering contrast, despite the similar hydrodynamic size. Overall, the scattering intensity of the liposomes is highly predominant with respect to all the other assemblies, and it dramatically decreases by addition of 1% Triton, due to complete liposome disruption and formation of mixed micelles, whose smaller size determines a lower scattering intensity. Concerning ENPs, the DLS profiles of 12-bis-THA 0.18 mM with 1% Triton can be reproduced as composed by the decays of ENPs and the detergent micelles, each weighted by the scattered intensity in the binary systems. Similarly, LNPs are not disrupted by the detergent, unlike what was observed upon sodium taurocholate addition. We hypothesize that the nanoplexes, with an overall positive charge, are dissociated by the negatively charged taurocholate amphiphile, which competes with TFD for association with 12-bis-THA. Figure 6b shows the normalized DLS curves of: liposomes, liposomes in the presence of ENPs and LNPs after 24 h incubation and for the same samples upon addition of 1% Triton. The DLS profiles of liposomes of the three formulations in the presence of both ENPs and LNPs perfectly overlap with those measured for native liposomes, highlighting that the overall liposomal structure is unaffected by the interaction with the 12-bis-THA assemblies, notwithstanding the increased membrane permeability of anionic liposomes upon interaction with LNPs. These results also mirror the observations made with bacterial cells, where the morphology of the bacterial cells was unperturbed. Upon addition of 1% Triton to liposomes-LNPs and liposomes-ENPs samples (after 24 h incubation), liposomes are completely disrupted while, as already discussed, both ENPs and LNPs are unaffected. The DLS profiles of liposomes-ENPs and liposomes-LNPs in the presence of Triton, can be therefore analyzed according to the presence of two distinct contributions to the total scattered intensity, the Triton-lipid mixed micelles (hydrodynamic diameter around 10 nm) and the residual ENPs or LNPs assemblies (hydrodynamic diameter around 200 nm). In Fig. 6c the normalized DLS curves of the liposomes-LNPs-Triton samples for the three liposomal formulations are compared. While the DLS profiles of the anionic liposomes are almost coincident, the slower decay of the POPC sample can be ascribed to the higher residual contribution of the LNPs, clearly more evident than that observed for POPC-PG and POPC-CL samples. Figure 6c reports the fitting results of the DLS profiles of the liposome samples, containing either ENPs or LNPs, after addition of Triton X100. The data were analyzed through Laplace inversion with CONTIN algorithm, which highlighted the presence of two populations, the mixed lipid/detergent micelles and the ENPs (or LNPs), each weighted by a coefficient, accounting for the respective contribution to the total scattered intensity. The contribution of Triton/lipid micelles with respect to ENPs is practically the same for the three formulations of liposomes, likely due to the low scattering intensity of ENPs as compared to the mixed micelles. Conversely, the relative contribution to the overall scattering intensity of the LNPs is meaningfully higher for the POPC formulation with respect to the two other liposomes containing CL or PG, as evident from the DLS profiles. These findings indicate that the interaction of LNPs with anionic liposomes causes a decrease of the concentration of the LNPs, which occurs to a lesser extent, in the presence of zwitterionic liposomes. Based on these results, we can hypothesize that the electrostatically-driven interaction between the LNPs and the anionic liposomes determines a strong binding of the LNPs with the bacterial membrane, leading to a structural destabilization of the liposomal membrane and simultaneous partial disruption of LNPs themselves.

**Figure 7:**
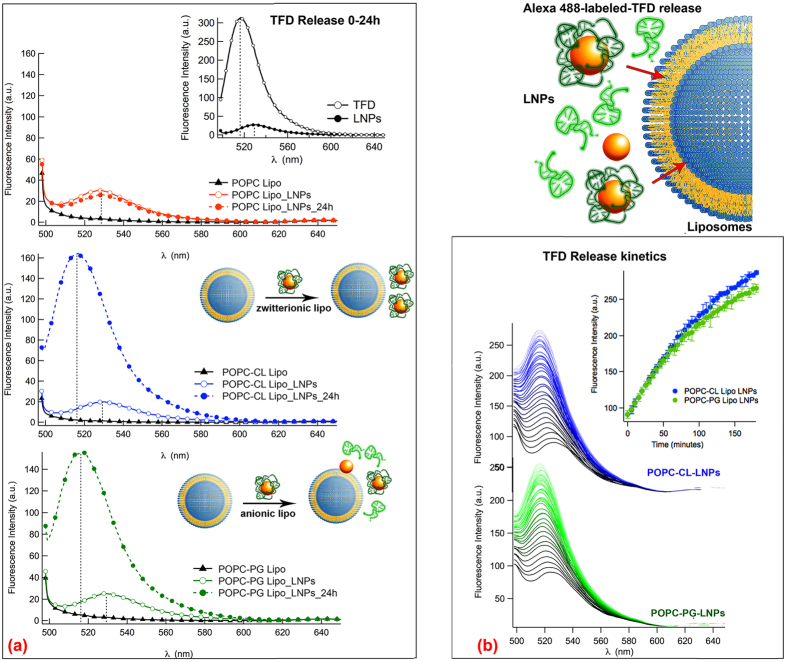
LNPs-liposomes interaction: TFD release. (**a**) (inset) Fluorescence spectra of Alexa 488 dye bound to TFD excited at λ excitation 488 nm alone (empty black market) and in the presence of 12-bis-THA (filled black markers); fluorescence spectra obtained at λ excitation 488 nm for bare liposomes (black lines and markers), and for liposomes (POPC, red lines and markers; POPC-CL blue lines and markers; POPC-PG green lines and markers) in the presence of LNPs containing Alexa 488 fluorescently-labeled TFD, 0 h (empty markers) and 24 h (filled markers) after preparation. (**b**) Alexa 488-TFD-LNPs fluorescence upon incubation with POPC-CL (blue lines) and POPC-PG (green lines) liposomes: fluorescence curves obtained at λ excitation 488 nm every 5 min and (inset) Alexa 488-TFD fluorescence recovery kinetics reported for POPC-CL (blue lines) and POPC-PG (green lines) liposomes.

Overall, the DLS results highlight the role of anionic (PG and CL) lipids, with respect to the zwitterionic ones in the interaction of the LNPs with the target membrane. However, a specific effect of CL with respect to PG is not clearly evident. To further address this point, and focus on the TFD fate upon interaction of the LNPs with the bacterial membrane models, we performed a fluorescence investigation on LNPs containing an AlexaFluor 488 (AF488)-labeled TFD. These fluorescent LNPs were added to liposomes of different composition. Figure 7a (inset) displays a representative fluorescence spectrum measured for the AF488 fluorescently labeled TFD alone (empty markers) and complexed by 12-bis-THA. This complexation causes a strong decrease of intensity and a shift in the fluorescence maximum from 516 nm to 529 nm. Thus, AF488 fluorescence quenching and red shift can be considered as fingerprints for TFD complexation by ENPs. Upon incubation of POPC liposomes with LNPs the fluorescence spectrum of AF488-TFD did not change, both in intensity and frequency, and was consistent with the sole presence of LNPs, even after 24 h. For liposomes containing CL (POPC-CL) or PG (POPC-PG), initially, no significant effect was observed. However, after 24 h for both samples a strong recovery of AF488 fluorescence intensity was observed, reaching around 50% of the intensity expected for free TFD. This evidence points out a release of the TFD upon incubation of LNPs with anionic liposomes. Finally, Fig. 7b compares the time courses of AF488-TFD fluorescence recovery, upon interaction with POPC-CL (blue) and POPC-PG (green) liposomes, over a three-hour period. Clearly, at variance with the behavior of POPC liposomes, the increase and shift of the fluorescence maximum observed both for POPC-PG and POPC-CL liposomes, reveals that the interaction between the LNPs and the anionic lipid membrane efficiently triggers LNP disruption (as also deduced from DLS results) and TFD release. If the emission intensities are plotted as a function of time (Fig. 7b), a slight but reproducible difference in the kinetics profiles can be observed for POPC-CL and POPC-PG, suggesting a slightly faster TFD release in the presence of CL.

**Figure 8:**
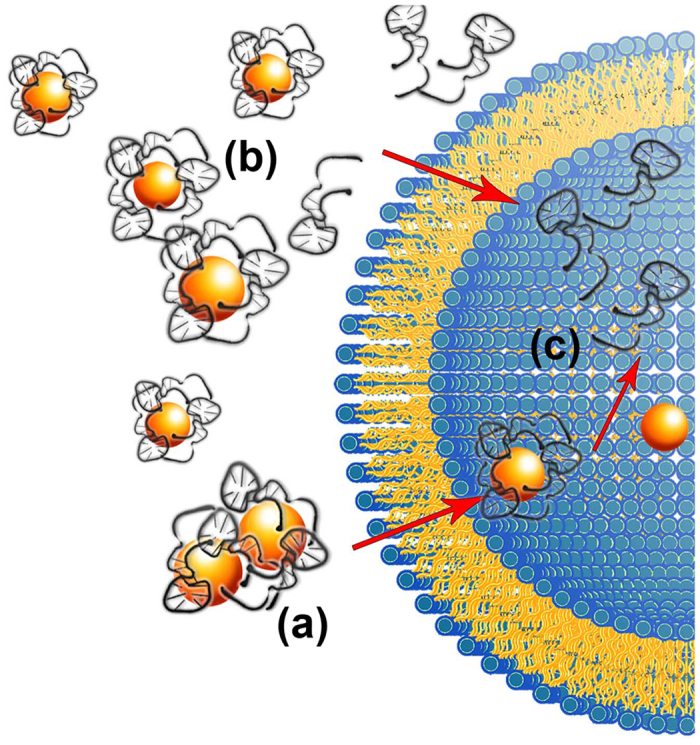
Hypothesized TFD delivery pathways inside *E. coli* cytoplasm. The electrostatically-driven interaction of the LNPs with the negatively charged bacterial membrane leads to the fusion of the LNPs with the bacterial membrane, with destabilization of the lipid membrane, in particular in the CL-rich regions and the contemporary partial disruption of the LNPs. Thus, entire LNPs (**a**) penetrate across the permeabilized bacterial membrane and subsequently release the TFD (**c**), or disrupted LNPs release the TFD that freely passes through the destabilized lipid membrane (**b**).

Overall, our experimental results on cell membrane model systems and on archetypal Gram-negative and Gram-positive bacteria, allows us postulating an interaction mechanism between the LNPs and the bacteria, and identifying some of the main factors involved in the antimicrobial efficiency of TFD-12-bis-THA nanoplexes, sketched in Fig. 8.

The electrostatic attraction between the cationic LNPs and the anionic lipids in the membrane is clearly the driving force for the docking of the LNPs on the bacterial membrane. This docking effect does not lead to the complete disruption of the lipid membrane, as highlighted both from the experiments on liposomes and on bacteria. However, it leads to a strong membrane destabilization in liposomal model membranes (that was highlighted as an increased membrane permeability to molecular species), possibly with formation of transient pores. We hypothesize that a similar effect occurs on bacterial membrane, i.e. a membrane restructuring of the bacteria upon docking of the LNPs, leads to an increase of the permeability to external species, eventually favoring the uptake of entire LNPs or free TFD inside the bacteria. On the other hand, the interaction of the LNPs with the bacterial membrane leads to the partial disruption of the LNPs and release of the TFD. In both processes, the electrostatic interaction and membrane destabilization have the key roles, and CL has peculiar features from both points of view. First, as already mentioned, CL is a bidentate anionic lipid, with a similar structure to that of two covalently bound PG molecules. Thus, it provides localized areas of highly negative surface charge density on the lipid membrane that can act as specific targets for the 12-bis-THA, which is a bidentate species as CL as well, with bipositive net charge; second, CL has a non-zero spontaneous curvature, which could turn the membrane fluctuations upon the docking of the LNPs on the bacterial membrane, into effective interaction or fusion points between the membrane and the 12-bis-THA assembly, leading both to membrane destabilization and to the disruption of the LNPs and TFD release. The efficient delivery of the TFD to the bacterial cells, as highlighted in the previously discussed experiments, can be connected to the data obtained on membrane models, to build-up a picture of a possible simplified mechanism of TFD delivery inside cells, as sketched in Fig. 8: first, the LNPs closely interact with the bacterial membrane, due to electrostatic compensation (Fig. 8a). The initial interaction is particularly favored in the CL-rich areas of the bacterial membrane, due to the specific physicochemical characteristics of CL. This interaction leads both to bacterial membrane destabilization and to partial release of the TFD, thus, the delivery of the TFD into the cytoplasm can occur either through a two-step process, where the LNPs pass through the membrane and then release the TFD (Fig. 8a–c) or through a one-step process, where the interaction of the LNPs with the bacterial membrane and the release the TFD inside the cytoplasm are associated (Fig. 8b). In both one-step and two-step delivery mechanisms, a key role of the prokaryotic specific anionic lipid CL is evident, and it can be attributed both to its high negative charge and to its peculiar structure.

## Conclusions

In this study we investigated the delivery mechanism of a novel antimicrobial system, made of nanoplexes of a TFD complexed with the cationic bolaamphiphilic 12-bis-THA. Confocal microscopy data on Gram-negative and Gram-positive bacterial models, were combined with Fluorescence and Dynamic Light Scattering data on liposomes of variable lipid compositions, taken as synthetic lipid bacterial membrane models. An unpredicted agreement of the results obtained with the different experimental techniques on model bacteria and on synthetic membrane models, allowed us identifying some key factors in the TFD delivery mechanism: the anionic lipids present in the bacterial membrane and in particular CL, which is accumulated in the pole and septum regions of the bacteria, drive the interaction between the nanoplexes and the bacterial membrane, possibly due to a combination of electrostatics and membrane destabilization/poration effects. Furthermore, we were able to hypothesize the main steps of the delivery process of the TFD-12-bis-THA nanoplexes, providing fundamental knowledge for the development of novel efficient TFD-based formulations of antimicrobial systems. Finally, MIC, MTT, haemolysis and *in vivo* efficacy studies show that the presented antimicrobial nanoplexes can be promising for the development of efficient therapeutics for the treatment of severe bacterial infections of both Gram-positive and Gram-negative bacteria.

## Methods

### Materials

All materials were purchased from Sigma-Aldrich unless stated otherwise.

### LNP preparation

The dispersion of 12-bis-THA with chloride counter ion (0.18 mM) in aqueous solution was obtained by first dissolving the compound in milliQ-water. The homogenous dispersion in water was then obtained by vigorous stirring with vortex, leading to the formation of empty nanoplexes (ENPs). Similarly, loaded nanoplexes (LNPs) were obtained by adding the bolaamphiphile dispersion (0.18 mM) to a solution containing the TFD in milliQ-water (10 μg/mL) and stirring the mixture with vortex for 30 seconds. When required, AF488-labelled TFD was used.

### Liposomes preparation

The proper amount of lipids was dissolved in chloroform. A lipid film was obtained by evaporating the solvent under a stream of nitrogen and overnight vacuum drying. The film was then swollen and suspended in warm (50 °C) milliQ-water (to obtain a final 5 mg/ml lipid concentration) by vigorous vortex mixing. For the carboxyfluorescein (CF)-based assays the films were suspended in warm 60 mM CF and then thoroughly dialyzed in milliQ-water using a 14 KDa-cut off cellulose membrane to remove all the non-encapsulated CF. In both cases, to prepare vesicles with narrow distribution, the dispersion was extruded 10 times through a 100 nm membrane as previously described^45^.

### SYBR Green assay

Encapsulation of DNA in the 12-bis-THA was assessed by a method based on a fluorescent DNA-dye^46^ with slight modifications. Briefly, 100 μl of each sample (ENPs, LNPs and appropriate controls) were pipetted into individual wells of a 96-well black plate (Costar, UK). Then, 10 μl of 2xSybrGreen I (LifeTechnologies, Paisley, UK), a green fluorescent DNA stain, were added into each well. In all cases, fluorescence was measured using a POLARstar Omega plate reader (BMG) with λ ex485/em520 nm settings.

### Agarose Gel electrophoresis

Samples of LNP were electrophoresed on a 1% agarose gel and post-stained with ethidium bromide. A DNase protection assay was performed by incubating a volume of LNP equivalent to 1 μg of DNA with DNAse I following manufacturer’s instructions and the extent of protection assessed by agarose electrophoresis. To ensure total release of the DNA and evaluate qualitatively its stability, disruption of the nanoplexes was obtained by addition of the surfactant sodium taurocholate (NaTc) at 50 mM final concentration. All samples were stored at RT for 144 h and then LNPs were treated with NaTc to induce TFD release. ENPs, LNPs and LNPs* were run in triplicates. ENPs controls were included to observe any non-specific interaction between the NPs and the dye.

### Confocal Microscopy

AF488-labelled DNA-LNPs (λ ex488/em519) were studied alongside ENPs controls. These formulations were diluted 2 fold with the corresponding bacteria in exponential growth phase culture (0.2–0.3 OD_630_). For samples containing *E. coli*, the membrane stain WGA-TMR (λ ex555/em580 nm, Life Technologies) was added to the samples at a final concentration of 10 μg/ml. The samples were incubated with constant agitation for 1–2 h at room temperature, protected from light in order to enable WGA labeling of the peptidoglycan layer. Then, smears were prepared by duplicate in poly-L-lysine coated microscopy slides and incubated for 1 h under the same conditions. The slides were gently washed twice with sterile PBS and air dried prior to mounting in Fluoroshield Mounting media. All samples were analyzed within 24 h of preparation. Samples were analyzed using a Leica SP5 confocal microscope using a 63× objective (Milton Keynes, UK). The images obtained were processed with the software Image-J (NIH Image). FM™ 4-64FX (λ ex563/em744 nm, Life Technologies) was employed as membrane stain for *C. difficile* 630. For health and safety reasons, *C. difficile* 630 cells were fixed with 3.6% formaldehyde while incubating on poly-lysine coated slides, followed by 2 additional PBS washes to remove the formaldehyde.

### Animal efficacy study

Golden Syrian Hamsters were used in a severe *C. difficile* infection (CDI) model. The animals were dosed with 30 mg/kg oral clindamycin 24 hours prior to infection with *circa* 100 *C. difficile* strain B1 spores by gavage to create an acute infection model of C. difficile infection, 4 hours prior to the commencement of treatments. There were 5 hamsters in each arm of the study and they were treated with 3 daily doses (with a period of 8 hours) delivered by oral gavage at 10 mL/kg over a period of 7 days. The four treatment groups were: vehicle (50 mM MES pH5.6), 25 mg/mL vancomycin, 2 mg/mL LNP with SigH TFD and 2 mg/mL LNP with a scrambled TFD. The primary endpoint of the study was survival ten days post infection, and in addition the *C. difficile* vegetative bacteria burden was measured in faecal samples from surviving animals. Statistical analysis employed Kaplan-Meier Survival analysis and Kruskal-Wallis: all pairwise comparisons (Conover-Inman post test) using Stats Direct or days survived data. All experiments were performed by Euprotec Ltd. (UK) in accordance with Animal (Scientific Procedures) Act 1986 (United Kingdom) and the animal protocols were approved by the University of Manchester ERP Committee.

### MTT assays

Caco-2 (ATCC HTB-37) were cultured at 37 °C in Dulbecco’s Modified Essential Medium (DMEM) supplemented with 10% heat-inactivated fetal bovine serum (FBS) and 2% penicillin-streptomycin. Cells were seeded in 96-well plates at 50,000 cells/cm^2^, left to grow until 40–50% confluency. After ~48 h media was removed and cells were incubated with 100 μl of serum-free media containing serial dilutions of the tested NP formulations. Cells were incubated for 48 h, then culture media was removed and MTT assay was performed. Briefly, cells were incubated with an MTT solution (400 μg/ml of MTT in media) for 2 hours at 37 °C. The MTT solution was then removed and 200 μl of DMSO was added and the 570 nm absorbance of the plates was read using a (BMG Labtech POLARstar Optima plate reader (Aylesbury, UK). Each experiment was performed three times.

### Haemolysis assay

Freshly harvested erythrocytes from Sprague Dawley rats were purchased as a 50% v/v suspension in Alsevers from Seralab (UK). Cells were centrifuged, resuspended in sterile HBSS to a volume of 2%. Aliquots (0.1 mL) of erythrocytes were added to round bottomed 96 well plates and mixed with an equal volume of serially-diluted ENP/LNP. Following incubation for 1 h at 37 °C under gentle agitation the plates were centrifuged at 1000 *g*. Supernatant (0.1 ml) was aspirated and applied to a fresh 96 well plate. The release of haemoglobin (A_550nm_) was measured on a Thermo Multiskan FC. Haemoglobin release was compared to 1% Triton X-100 (100% release) and HBSS (0% release).

### Fluorescence spectrofluorimetry

Fluorescence measurements were carried out on a LS50B spectrofluorimeter (Perkin Elmer). Liposomes (100 μl) were added into 500 μl milliQ-water and the background fluorescence was measured. Then, 100 μl of either ENPs or LNPs were added, incubated for 5 minutes (for CF release experiments) and for 5 minutes and a second time point at 24 h (for AF488 release experiments). For AF488 release experiments kinetic curves were also acquired, in a 3 h time span. To disrupt the liposomes as a positive control for CF release, Triton X-100 at 0.5% final concentration was used. In all cases the settings used were as follows: λ emission was acquired from 498 to 650 nm with λ excitation 488 nm (both for AF488-labelled DNA and for CF).

### Zeta Potential

All Zeta potential measurements were taken using a zeta potential analyzer (Zeta Plus, Brookhaven Instruments Corporation, Holtsville, NY). Zeta potentials were obtained from the electrophoretic mobility *u*, according to Helmholtz–Smoluchowski equation:


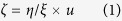


with *η* being the viscosity of the medium and *ε* the dielectric permittivity of the dispersing medium. The zeta potential values are reported as averages from 5 measurements on each sample.

### Dynamic Light Scattering (DLS)

DLS measurements were carried out on a Brookhaven Instruments apparatus (BI 9000AT correlator and BI 200 SM goniometer). The signal was detected by an EMI 9863B/350 photomultiplier. The light source was the second harmonic of a diode Nd:YAG laser, λ = 532 nm Coherent DPY315M-100, linearly polarized in the vertical direction. The normalized electrical field time autocorrelation functions of the normalized intensity time autocorrelation of the scattered light were measured at 90° and analyzed according to the Siegert relationship, which connects the first order or field normalized autocorrelation function *g*_*1*_(*q, τ*) to the measured normalized time autocorrelation function *g*_*2*_(*q, τ*):


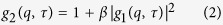


with *β* being the spatial coherence factor which depends on the geometry of the detection system. The functions (*β|g*_*1*_(*q, τ)|*^2^)^*1*/*2*^ were also normalized to vary between 0 and 1 for display purposes. The field autocorrelation functions were analyzed through the cumulant fitting stopped to the second order for samples characterized by a single, monodisperse population, allowing an estimate of the hydrodynamic diameter of particles and of the polydispersity index. For polydisperse samples (i.e. ENPs and LNPs incubated liposomes after treatment with Triton), data were analyzed through the Laplace inversion according to CONTIN algorithm.

## Additional Information

**How to cite this article:** Marín-Menéndez, A. *et al*. Antimicrobial Nanoplexes meet Model Bacterial Membranes: the key role of Cardiolipin. *Sci. Rep.* **7**, 41242; doi: 10.1038/srep41242 (2017).

**Publisher's note:** Springer Nature remains neutral with regard to jurisdictional claims in published maps and institutional affiliations.
